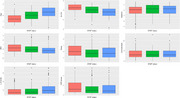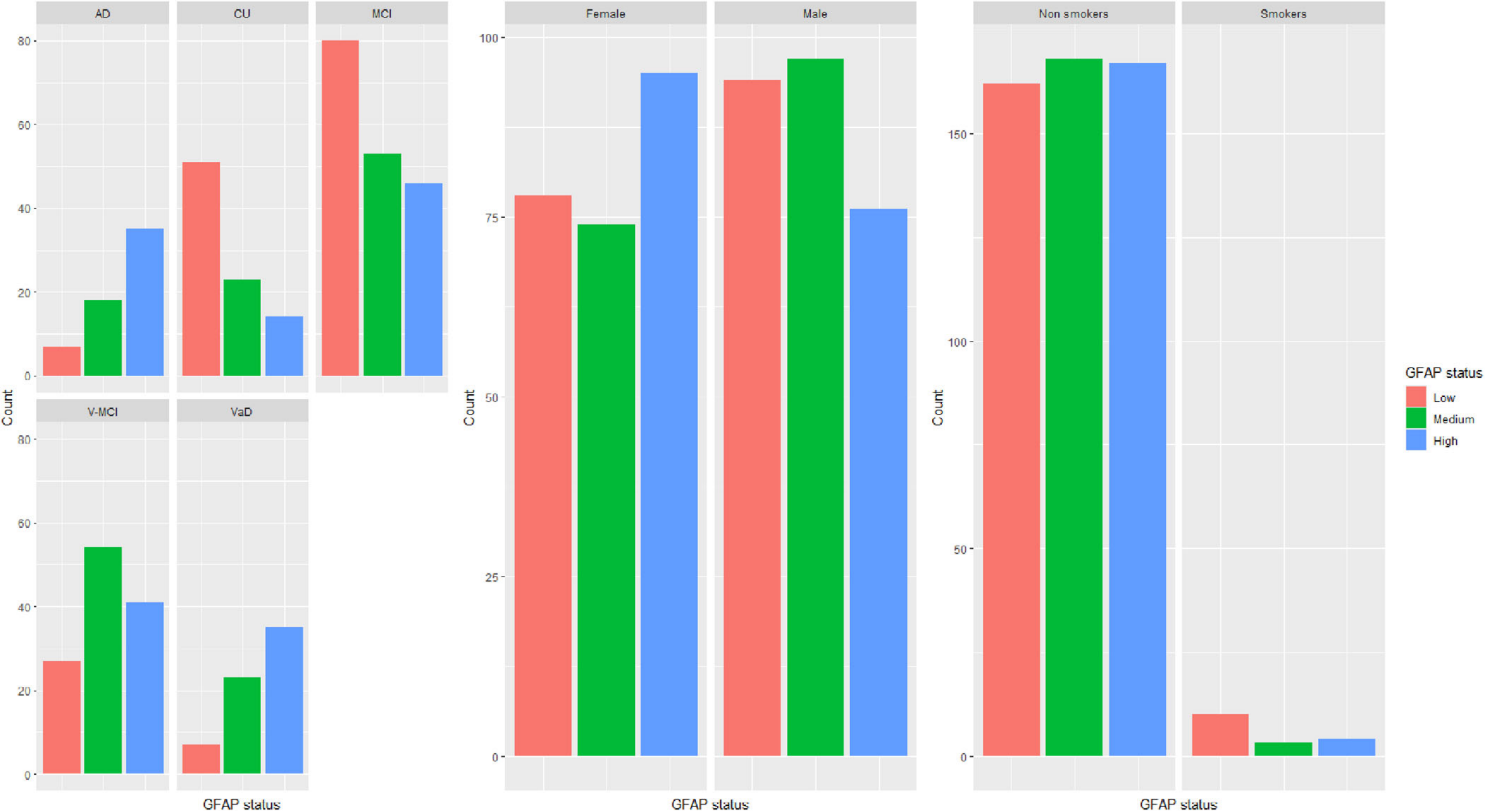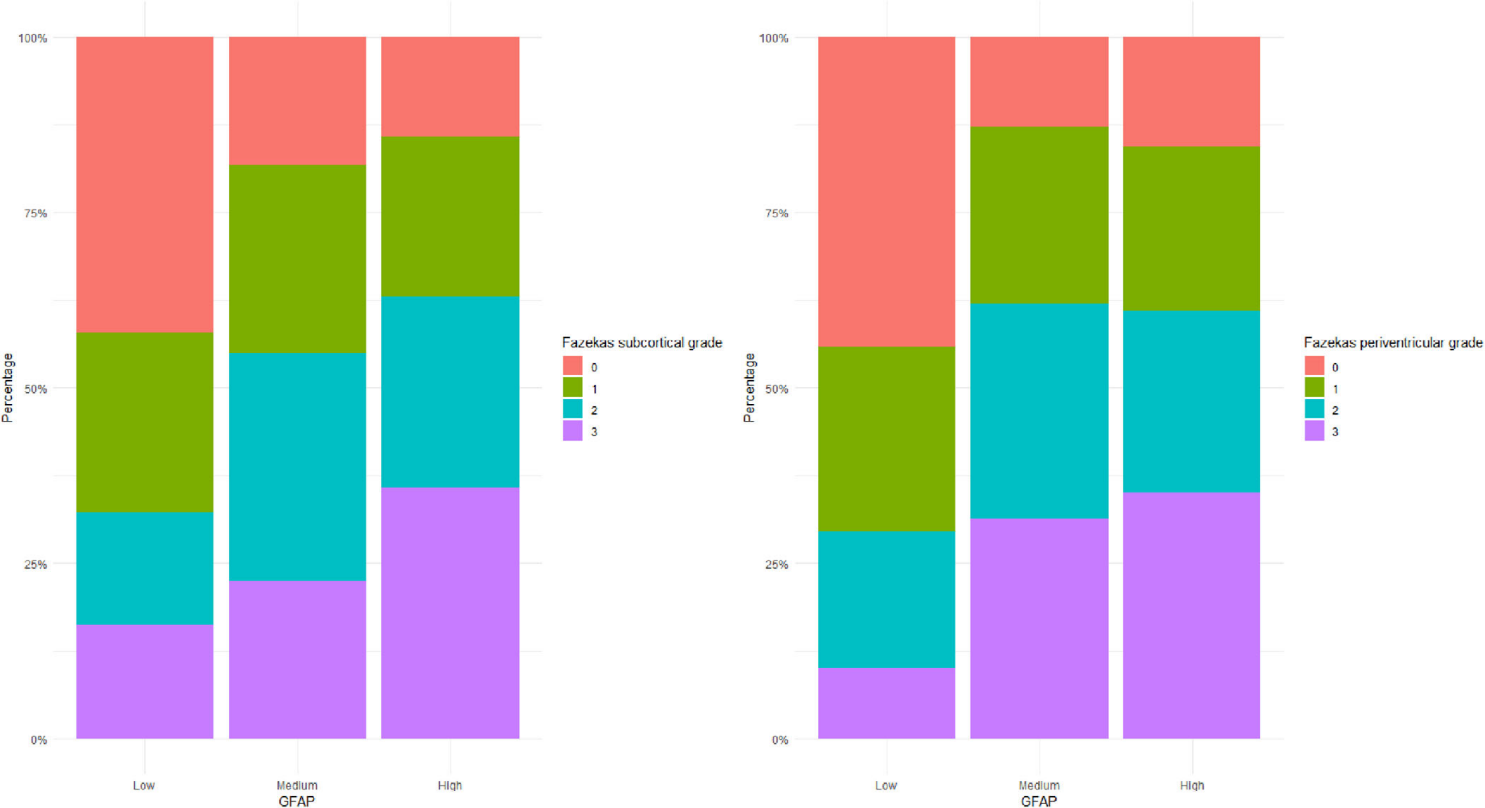# The relationship between neuroinflammation and clinical features of dementia: An investigation of GFAP levels in Alzheimer's disease and related disorders

**DOI:** 10.1002/alz70856_098224

**Published:** 2025-12-24

**Authors:** Bruna Seixas Lima, Pedro Rosa‐Neto, Durjoy Lahiri, Carlos Tyler Roncero, Howard Chertkow

**Affiliations:** ^1^ Baycrest Academy for Research and Education, Toronto, ON, Canada; ^2^ McGill University, Montreal, QC, Canada; ^3^ Department of Medicine, Queen's University, Kingston, ON, Canada

## Abstract

**Background:**

Neuroinflammation has been recognized as an important element in Alzheimer's disease (AD) and related dementias. Glial fibrillary acid (GFAP) is an astrocytic cytoskeletal protein that reflects neuroinflammation. Our study investigated levels of GFAP in serum and clinical features of individuals with AD, vascular dementia (VaD), mild cognitive impairment (MCI), and vascular MCI (V‐MCI) in comparison to a cognitively unimpaired (CU) group. Our goal was to reveal the relationship between neuroinflammation and clinical features of these groups, such as degree of brain vascular disease.

**Methods:**

We investigated individuals with AD (*n* =  60); VaD (*n* = 65), MCI (*n* = 179), V‐MCI (*n* = 122) and cognitively unimpaired controls (CU; *n* = 88). Normal GFAP range for older adults has been suggested as 40.7–228 pg/ml. We divided participants into tertiles categorized as low (≤113.05 pg/mL), medium (113.05–184.9 pg/mL), and high (185 – 461 pg/mL). Cumulative link modeling (CLM) was used to investigate the relationship between the GFAP and age, sex, diagnostic group, history of smoking, number of comorbidities (those associated with inflammation), body mass index (BMI), nutrition score, sleep quality index, peripheral inflammation – measured by Interleukin‐6 (IL‐6) and C ‐ reactive protein (CRP) levels, and Fazekas scale for a measure of periventricular and subcortical white matter damage on MRI.

**Result:**

We found significant main effects between GFAP groups and age, diagnosis of AD, diagnosis of VaD, MoCA, sex, CRP and BMI. Pairwise comparisons revealed significant differences between all GFAP groups, age, and MoCA scores. Low and medium GFAP levels were related to CRP levels. Low and medium, and low and high GFAP levels were related to BMI. No differences were found between GFAP status and nutrition, sleep, smoking, number of comorbidities, Fazekas scale and diagnosis of MCI or V‐MCI.

**Conclusion:**

Neuroinflammation (GFAP levels) increases as individuals age, but to a greater extent in the presence of cognitive impairment (especially AD and VaD groups). Levels of peripheral inflammation (CRP) and BMI, were inversely associated with neuroinflammation. Elevated GFAP levels were not related to increased MRI white matter lesions or other elements of vascular disease. Further studies will investigate these complex relationships.